# Cardiac Output Monitoring in Preterm Infants

**DOI:** 10.3389/fped.2018.00084

**Published:** 2018-04-03

**Authors:** Matthew McGovern, Jan Miletin

**Affiliations:** ^1^Neonatology Department, Coombe Women and Infant University Hospital, Dublin, Ireland; ^2^Department of Paediatrics, Trinity College Dublin, National Children’s Hospital Tallaght, Dublin, Ireland; ^3^Institute for the Care of Mother and Child, Prague, Czechia; ^4^3rd School of Medicine, Charles University, Prague, Czechia; ^5^UCD School of Medicine and Medical Sciences, Dublin, Ireland

**Keywords:** preterm, neonate, cardiac output, hemodynamic, monitoring, perfusion

## Abstract

Maintaining optimal circulatory status is a key component of preterm neonatal care. Low-cardiac output (CO) in the preterm neonate leads to inadequate perfusion of vital organs and has been linked to a variety of adverse outcomes with heightened acute morbidity and mortality and adverse neurodevelopmental outcomes. Having technology available to monitor CO allows us to detect low-output states and potentially intervene to mitigate the unwanted effects of reduced organ perfusion. There are many technologies available for the monitoring of CO in the preterm neonatal population and while many act as useful adjuncts to aid clinical decision-making no technique is perfect. In this review, we discuss the relative merits and limitations of various common methodologies available for monitoring CO in the preterm neonatal population. We will discuss the ongoing challenges in monitoring CO in the preterm neonate along with current gaps in our knowledge. We conclude by discussing emerging technologies and areas that warrant further study.

## Introduction

Monitoring and maintaining adequate cardiac output (CO) is a key component of cardiovascular care in the preterm neonate. Low-output states have been associated with a variety of adverse outcomes and there is some evidence that low-central blood flow may respond to medical therapy (1, 2). Immaturity of the cardiovascular system predisposes the preterm neonate to low-flow states and relative immaturity of other organ systems means that premature infants are vulnerable to organ damage as a result of low flow. The unique anatomy and physiology of preterm infants makes monitoring of CO a difficult process, complicated by the transitional circulation and the presence of shunting.

Abnormal perfusion is recognized as having adverse effects on the preterm neonate with reduced mean arterial pressure being associated with increased mortality, increased severe intraventricular hemorrhage (IVH) and ischemic brain lesions (3). Duration of hypotension also correlates with developmental outcome in very low-birth weight infants (4), and extremely low-birth weight infants with treated hypotension are at risk of hearing loss, motor delay, and death (5). While such derangements in traditional clinical measurements of perfusion clearly have prognostic implications for the neonate, traditional clinical assessment is known to be of limited use in predicting central blood flow within the pediatric population (6). Blood pressure is one of the commonest clinical methods of assessing circulatory status; however, accurate measurement in the preterm population is difficult and there is no consensus definition on hypotension. In addition, blood pressure shows poor correlation with central blood flow (7–12) and is likely to be a late sign of uncompensated low-perfusion meaning it is an insensitive sign in early circulatory compromise. Capillary refill (7, 8, 13), urine output (7), and temperature (8) are similarly unreliable for detection of low perfusion in the preterm neonatal population. While in combination, these clinical signs are undoubtedly useful in defining critically unwell infants they are clearly inadequate as markers of perfusion in the preterm population as they lack sensitivity in the early stages of disease where medical intervention is likely to have the greatest role.

As a result, neonatologists should turn to more objective measurements in assessment of perfusion within this population. The availability of bedside measures of CO such as echocardiography is acknowledged as an important tool for adult and pediatric intensivists in improving outcome (14). The non-invasive nature of echocardiographic measurements along with the real-time information provided means that they are favored in the acute setting, and there is evidence that the availability of bedside CO monitoring positively impacts patient care (14–17). Low-central blood flow measurements in preterm infants are associated with a variety of early adverse outcomes including altered electroencephalographic activity, oliguria, hyperkalaemia, necrotizing enterocolitis, retinopathy of prematurity, IVH, and death (8, 18–25). Preterm infants with reduction in left ventricular output (LVO) or right ventricular output (RVO) of more than 50% in late-onset sepsis have increased mortality (26) and low-central blood flow has been linked to adverse long-term neurodevelopmental outcomes in the preterm population (20, 27). Low CO is also a common perioperative complication for children with congenital heart disease (28), and is a risk factor for prolonged mechanical ventilation following cardiac surgery (29) and for adverse neurodevelopmental outcome (28).

Given the implications of reduced central blood flow in the preterm neonate, there is a need for a robust, non-invasive, and continuous measure of CO within this population. Criteria for an ideal technology have been outlined in previous publications (30), though at present no ideal technology exists.

## Traditional Invasive Methods

Due to the availability of newer, less invasive technologies many of the “gold standard” invasive techniques used in adult medicine are rarely used in the neonatal population. These methods still merit discussion as they are held by some as the most accurate method of evaluating CO despite their infrequent clinical use and limited data on repeatability within the pediatric population. One of the oldest methods of invasively measuring CO are techniques based on the Fick principle. The Fick principle measures blood flow to an organ (most usually used to measure systemic blood flow as a whole) based on the idea that the blood flow may be calculated if the amount of a substance taken up by an organ over time is known, and the quantity of the substance can be measured both proximal to and distal to the organ of interest. The classic methodology used oxygen and stated that CO may be calculated if oxygen consumption, arterial oxygen concentration, and venous oxygen concentration are known. Subsequent adaptations of the Fick principle have also used carbon dioxide, with animal models suggesting that the methodology may be potentially viable in the neonatal population (31). The Fick methodology has been used successfully in term neonates (32, 33) and appears to correlate well with other invasive methodologies within the pediatric population (32). The obvious disadvantages of this methodology are the requirement for arterial and venous lines and the need for accurate breath-by-breath oxygen consumption calculation which is likely to prove difficult in preterm neonates where the commonly used uncuffed endotracheal tubes are likely to make such measurements inaccurate.

Modern thermodilution techniques rely on placement of a specialized catheter within the pulmonary artery with a temperature probe placed distally. The most commonly employed example is the Swan-Ganz catheter, placement of which has previously been undertaken successfully in the preterm neonatal population (34). This catheter has a temperature probe at the tip and at a proximal point which lies in the right atrium there is a port through which a cold solution is injected. Following injection of a cold solution into the right atrium, the catheter tip in the pulmonary artery detects the temperature change relative to the dilution within the blood allowing accurate measurement of CO. The original description of the Swan-Ganz catheter showed that it produced comparable values to dye-dilution with a repeatability of 4.1% (35). While held as the “gold standard” by many there are a variety of potential pitfalls to the technique (36, 37). Variations on this technique including trans pulmonary thermodilution have been developed (38), and are feasible in the pediatric population with high repeatability (39). Despite evidence of validation in comparison with the Fick methodology in children (33), data in the preterm neonatal population is limited, and the technique is seldom used due to technical restraints.

Dye-dilution is based on injection of a dye in the pulmonary artery and measurement of the dye concentration through peripheral arterial line. If the concentration and volume of dye injected is known, CO is subsequently calculated based on the concentration detected peripherally over time (40). Experiments in humans have confirmed that the dye-dilution methodology correlates well with the Fick methodology and thermodilution (41). Modifications of this technique have been utilized in the term neonate to determine CO (42), but similar to thermodilution this has not entered routine practice and has not been examined in the preterm neonatal population.

While there is no true “gold standard” for CO monitoring in the neonatal population, invasive technologies are considered by many to be the most accurate methodologies for determining CO. Their clinical use in the neonate is rare due to a variety of important limitations. Infection, damage of surrounding tissue, and thrombus formation number among the most serious complications of central catheter placement. Other difficulties arising from use of the techniques include the need for arterial and venous blood sampling and potential volume overload from injection of substrate. As well as the risk of morbidity and technical limitations, catheter placement is a source of discomfort and the measurements of output, while likely accurate, are not continuous.

## Minimally Invasive Techniques

Pulse contour and pulse power analysis make inferences about CO and other central blood flow parameters based on a peripherally measured arterial pulse wave. Both techniques rely on the presence of a peripheral arterial line and these techniques represent two of the most extensively investigated minimally invasive technologies for measuring CO in the adult population. Pulse contour analysis relates the contour of arterial pressure over time to stroke volume and systemic vascular resistance. A mathematical algorithm is then used to calculate CO based on measurements taken from a blood flow sensor within a peripheral artery and a variety of devices such as the FloTrac, pulse index continuous cardiac output monitor, and pressure recording analytical method represent variations on this idea (43). Pulse contour analysis has been used successfully in older children with congenital heart disease (44), but concerns have been raised with regards to its accuracy in relation to traditional methodologies within the pediatric population (45). Pulse power analysis is a similar technology relying on peripherally obtained arterial measurements to deduce central flow measurements. Pulse power analysis relies on the idea that variations in pulse power detected peripherally are equivalent to stroke volume minus the blood volume sent to the periphery of the body. As the title suggests, the power of the arterial pulsation rather than its contour is used to calculate CO using a mathematical algorithm. Pulse power devices have not been extensively investigated in pediatric patients to date. While these devices are minimally invasive, there are a variety of downsides to using this technology (46). Changes in systemic resistance and certain cardiac conditions are known to affect the results obtained in adults, and while minimally invasive, certain devices require calibration before use and both methodologies rely on placement of an arterial catheter.

Partial gas rebreathing based on a modification of the Fick principle has emerged as a much less invasive alternative for measuring CO in adults but remains untested in preterm neonates. Ultrasound dilution methodology relies on changes in ultrasound velocity within blood following injection of body-temperature isotonic saline to calculate CO. It has compared favorably to more traditionally invasive techniques in older children (47, 48), and *in vitro* work has shown that the technology may be feasible in neonates (49). Despite some promising results, ultrasound dilution requires placement of arterial and central venous catheters to create and extracorporeal loop and has not been validated *in vivo* in the neonatal population to date. Lithium dilution is a method of calculating CO where a Lithium Chloride solution is injected through a central venous catheter and a sensor on a peripheral artery measures the lithium concentration over time to estimate CO (50). Safety and accuracy when compared with thermodilution have been established in a pediatric population including some neonatal patients (51), but this technology has not been evaluated in preterm infants to date.

## Non-Invasive Methodologies

### Echo-Based LVO

Left ventricular output measurement by echocardiography represents the outflow of oxygenated blood from the left side of the heart. In the absence of shunting LVO represents systemic blood flow and hence cumulative blood flow to all major organs. In theory, changes in LVO reflect changes in blood flow to the periphery of the neonate. LVO is measured according to the following formula:

$$\mathrm{LVO}\;(\text{mL}/\text{kg}/\text{min})=\frac{\begin{array}{l} {}[\mathrm{Velocity time integral}\;\times \;\mathrm{Cross sectional area}\;\\ \;(\mathrm{aortic valve})\;\times \;\mathrm{Heart rate}]\; \end{array}}{\mathrm{Weight}(\text{kg})}$$

Annulus size is measured at one of three locations: between the aortic valve hinges, at the aortic sinus, and at the sinotubular junction using a parasternal long-axis view. It has been suggested that the sinotubular junction may be the most accurate method of measuring diameter (52), however, no gold standard approach exists and most guidance suggests measurement just below the aortic valve (53). Cross-sectional area is calculated at the level of the aortic valve using the long parasternal view at the end of systole, and velocity time integral (VTI) is measured using pulse wave Doppler just proximal to the aortic valve in the apical five-chamber view (54). As with all Doppler techniques, the angle of insonation has potential to effect the accuracy of all echocardiography techniques. This is a particular issue in the measurement of LVO where the angle of insonation is known to be larger on the left outflow tract than on the right (55). Most modern echocardiography equipment incorporate software correction to combat this, however, this correction is not without issues and some clinicians prefer to use the initial uncorrected measurements if the angle of insonation is <20°. In experienced hands, LVO represents an easily performed measure of blood flow to the periphery of the baby and as with all echo measurements has the advantage of providing real-time measurements of systemic blood flow facilitating rapid decision making in the neonatal intensive care unit (NICU). The technique is well-established in the pediatric population and was first described in preterm infants over 30 years ago (56). Early work showed that LVO correlated well with traditional cardiac catheterization and thermodilution in term neonates (57), though it is worth noting that infants used in early studies were generally outside the transitional period. Despite these advantages, there are several limitations to the use of LVO in the preterm population. LVO calculation relies on calculation of vessel diameter, hence any inaccuracy in measurement will be magnified when vessel diameter is squared during the cross-sectional area calculation (54). Doppler-derived CO measurement in the pediatric population has traditionally been prone to considerable inter- and intra-observer variability (58), however, recent studies on repeatability within the preterm neonatal population are lacking. Studies looking at repeatability in neonates have found that in experienced hands intra-observer variability for LVO can be as low as 3.6% (59), and that inter-observer variability may not differ significantly between users (60). While improved training and technology have undoubtedly lessened the potential bias of measurements between and within users, the potential for such differences cannot be discounted and could contribute to clinically meaningful differences. Foremost among the limitations of LVO in the preterm population is the impact of shunting on the LVO measurement (61), with expert guidance stating that LVO does not represent systemic blood flow in the presence of a patent ductus arteriosus (PDA) (53). Since a PDA is present in most preterm neonates the technique has limited value as an isolated measure of systemic perfusion in this population but may be of use in combination with other parameters as a global overview of preterm circulatory status. It is likely that the greatest precision in functional echocardiographic measurements will come with repeated measures taken by the same experienced user and as discussed elsewhere, functional echocardiography should not rely on a single measurement, but rather on repeated measurements over time to assess changes in circulatory status.

### Echo-Based RVO

Right ventricular output represents another commonly recorded measure of central blood flow. In the absence of shunting, RVO reflects the cumulative inflow of deoxygenated blood and hence venous return. RVO is measured according to the following formula:

$$\mathrm{RVO}\;(\text{mL}/\text{kg}/\text{min})=\frac{\begin{array}{l} {}[\mathrm{Velocity time integral}\;\times \;\mathrm{Cross sectional area}\\ \;(\mathrm{at pulmonary valve})\;\times \;\mathrm{Heart rate}]\; \end{array}}{\text{Weight }(\mathrm{kg})}$$

Cross-sectional area is calculated at the level of the pulmonary valve at the end of systole using an oblique long parasternal view or short parasternal view, and VTI is calculated just proximal to the pulmonary valve in the same views (54). Measurements of vessel diameter and VTI for RVO have traditionally both been obtained in this manner, however, recently published material has suggested several adaptations to standard right ventricular imaging protocols to address the unique issues in echocardiography within the neonatal population during the transitional period (62). As a result, there will likely be increasing use of additional views such as the right ventricle 3-chamber view in assessing right ventricular outflow and function in future publications. Similar to left ventricular measures, RVO was first described in the neonatal population approximately 30 years ago (63, 64). Because the reference points used in measurement lie close to the anterior chest and the view for calculating VTI and cross-sectional area are the same, RVO is easily measured in the preterm infant. Like all measurements taken with echocardiography, RVO provides results in real-time and is non-invasive. No information is available on the accuracy of RVO in relation to invasive methods in the preterm neonatal population. Similar to LVO, RVO is affected by the presence of shunting, in this case a patent foramen ovale or other septal defects leading to inaccuracy in measurement (61). The issue of inter and intra-observer variation in measurement is also present in RVO measurement with repeatability being similar to measurement of LVO in the term and preterm population (60). In a recent study by Popat et al., differences in measurement led to significantly different results between users with the majority of the difference being made up by inaccurate measurement of VTI and vessel cross-sectional area (65). Because of the limitations of RVO, the technique has a limited role in the assessment of the circulatory status of preterm infants. Similar to other echo techniques, the maximal benefit is likely to come from recording RVO along with other complementary echo measurements, with readings repeated over time and ideally taken by the same user to reduce variation.

### Superior Vena Cava (SVC) Flow

Due to the limitations of LVO and RVO measurement within the neonatal population SVC flow was developed as an alternative measure of central blood flow which is not affected by the presence of shunting (66). The flow within the SVC is calculated according to the following formula:

$$\text{SVC flow }(\text{mL​​}/\text{​kg​}/\text{​min})=\frac{\begin{array}{l} \;[\text{Velocity time integral }\\ \times \;(\pi \times ({\text{Mean SVC diameter}}^{2}\text{​}/\text{​}4)\;\times \text{ Heart rate})] \end{array}}{\text{Weight }(\text{kg})}$$

The diameter of the vessel is measured *via* the high-parasternal view as the vessel enters the right atrium with VTI traditionally calculated from a low-subcostal view as the SVC enters the right atrium (66). Though this is the most common approach, a recent publication suggests that measurement of vessel area from a short axis view and VTI from a suprasternal view may improve repeatability (67). Since its initial description, low-SVC flow in the neonatal population has been shown to correlate with a variety of adverse short- and long-term outcomes (8, 9, 18–23, 27, 68). Compared with the other echocardiography-based techniques described, SVC flow has the advantage of being unaffected by shunting and is considered by many to be the most robust echocardiography technique for measurement of central blood flow (69). Similar to other methodologies discussed, however, there are concerns around the repeatability of the technique with studies on inter- and intra-observer variability, showing potential variability of between 1 and 102% and 1 and 34%, respectively (66, 70, 71). Much of the variability is attributed to probe positioning and differences in SVC diameter measurement (65), which are a particular problem given the compressible nature of the SVC’s venous wall.

### Bioreactance and Bioimpedance

At its most basic level, the thorax may be seen as a 3D shape with fluid (blood) flowing through it. Depending on the point in the cardiac cycle, the amount of fluid in the thorax will vary, for example, during systole blood flow through the aorta will increase. Different substances within the chest have different resistances to electrical flow, with blood having lower resistance than soft tissue or bone. Because blood flow changes based on CO between heartbeats, the relative resistance to electrical flow will alter depending on flow through the aorta. Bioimpedance uses this principle to measure CO by passing an electrical signal of known amplitude and frequency between electrodes across the chest and measuring the resistance to flow. With more blood flow in the aorta, the resistance to the passage of electrical signal is lower as calculated by the ratio of voltage to current amplitude. Hence, differences in resistance can be used to estimate CO. While traditional bioimpedance is continuous, non-invasive, and requires relatively little expertise to perform; it is limited by the interference from electrical noise, the need for exact placement of electrodes for accuracy and concerns over accuracy when compared with more established techniques in the pediatric population (72). An adaptation of bioimpedance called electrical velocimetry has been suggested by some as improving the accuracy of the technique and has been shown to be feasible in the neonatal population (73). In addition, CO measurements taken by electrical velocimetry appear to compare favorably with echocardiography measures in children with congenital heart disease (74), term neonates (75), and within the preterm population (76). Despite these advantages, the bioimpedance-based methodology has not yet been adequately validated in the neonatal population either against traditional invasive methodologies or with regard to clinical outcomes.

Another new methodology based on measures of thoracic electricity conduction is bioreactance, which measures the phase shift of an electrical signal as it passes through tissues and is thought to be less susceptible to noise interference than traditional bioimpedance (77). Bioreactance relies on the fact that changes in phase shift can only occur in the setting of pulsatile flow and given that the majority of pulsatile flow within the thorax is accounted for by the aorta, phase shift will reflect flow in the aorta and hence CO (78). Similar to bioimpedance, electrodes are placed on the chest for measurement. In bioreactance electrodes on the upper and lower chest measure bioreactance on the left and right sides of the chest separately and the results are averaged to determine CO. Despite initial concerns regarding the accuracy of the technique in smaller children (79), bioreactance has been shown to be feasible in both the term and preterm population (80, 81). There is also evidence that bioreactance has shown some potential to monitor fluid status in postoperative pediatric patients (82). While measurements of LVO by bioreactance have been shown to correlate with echocardiography measurements, bioreactance appears to underestimates CO (80). Bioreactance is similarly non-invasive to bioimpedance but has the advantage of not being dependant on distance between electrodes and is less affected by electrical “noise,” a common feature in the NICU setting. While bioreactance is widely considered a more robust technique, it has not yet entered routine use in the neonatal population and there are concerns from adult studies regarding its accuracy in low-flow states.

Despite the limitations of both bioimpedance and bioreactance methods, the only existing systematic review and meta-analysis of the accuracy and precision of non-invasive CO techniques in the pediatric population concluded that electrical cardiometry was the most accurate, with other methodologies varying greatly between studies (83). At present, both techniques are used primarily in a research capacity but represent two of the most promising devices for the non-invasive measurement of CO in the preterm neonate.

### Portable Doppler

Devices such as the ultrasonic cardiac output monitor (USCOM) monitor measure Doppler flow within the large vessels of the chest transthoracically. The technique relies on external placement of a small Doppler probe, which when angled correctly can measure the flow within the aorta and calculate CO based on an algorithm which uses patient height to estimate cross-sectional area. The technique is non-invasive, continuous, and easy to learn (84). The technique has been compared with thermodilution in the pediatric population with results suggesting that the technique was inaccurate for estimation of actual CO measurements (85). The technique has been used successfully within the preterm neonatal population and shows good correlation with echo measurement of CO (59, 86) though due to the small number of studies in the preterm population there are ongoing concerns about the accuracy of the technique in neonates (87). One study looking at the repeatability of USCOM has suggested that a variety of factors make measurement easier in younger patients (88), which may favor the technique in the pediatric and neonatal population. Despite promising data from adult patients (89), the only meta-analysis assessing accuracy and precision of non-invasive CO measures in children suggested that Doppler flow techniques are prone to a high-percentage error (83). Esophageal Doppler is a related technique which is used successfully in the adult population. Because this methodology involves placement of a Doppler probe within the esophagus to measure flow in the descending aorta, it is limited by the size of the child and only used in infants >3 kg (90), making it of limited use in the preterm neonatal population.

### Magnetic Resonance Imaging (MRI)

Magnetic resonance imaging has safely been used in the preterm neonatal population to derive central blood flow measurements (91). MRI has been suggested as having improved accuracy and repeatability compared with other techniques for CO measurement in neonates and older children (92, 93). The obvious disadvantages of MRI are that the technique is slow, expensive, non-continuous, not routinely available to neonatologists, and non-portable. The technique has also not yet been compared with traditional invasive techniques in the neonatal population. As a result, MRI is not ideal as a methodology of assessing CO in preterm neonates as it cannot be performed at the bedside to facilitate decision making in critically unwell infants. Consequently, at present CO from MRI is limited to experimental use but may have a role in the development of future techniques for CO assessment or to improve existing technologies.

## Evidence for Improved Outcome with CO Monitoring

As the use of functional echocardiography has increased in the NICU so too have efforts to standardize the quality of imaging obtained in different centers. In addition, there is a limited but increasing body of evidence that monitoring CO in the preterm population improves outcome. While this article focuses on the use of point of care ultrasound for assessing CO, there are many other ways in which functional echocardiography can be used to improve neonatal care (94). It has been suggested that the availability of CO monitoring such as functional echocardiography can improve the care of both term and preterm infants in a variety of ways (95, 96), though as previously discussed there is only a limited pool of evidence for this to date. Despite this, there is data supporting the role of CO monitoring in clinical decision making and in some cases avoiding unnecessary intervention in the neonatal population (97). Functional echocardiography measurement of LVO allows early detection of cardiorespiratory instability in neonates with PDA ligation (98), a situation which is potentially amenable to medical intervention. Novel functional echocardiography measures also have potential to provide valuable information on myocardial performance following ligation of ductus arteriosus (99). In addition to targeted imaging on the basis of a known circulatory issue, functional echocardiography has also potential to screen preterm infants for asymptomatic but potentially clinically important abnormalities which are not uncommonly uncovered during routine imaging (100, 101).

## Limitations and Current Situation

Neonatologists currently stand in an unusual position with regard to CO monitoring: there are numerous techniques available for the evaluation of CO in the neonatal population but few have been rigorously validated against the classically held “gold standards.” Fewer still have been validated against clinical outcomes and we are often unsure if intervention to improve the measurements taken is improving the outcome of the infant. Like many aspects of neonatology, rigorous validation of new CO technologies is challenging as the traditionally held best practice, in this case invasive dilution techniques, have themselves been largely extrapolated from adult studies and are of uncertain utility in neonatology. Added to this is the inherent difficulty of defining a “normal range” within the neonatal population as identifying what constitutes a “healthy” preterm infant is not easy. The solution may seem obvious to some: designing randomized trials where some infants are assigned to receive a novel investigation and some are not. On the surface, this seems easy but there are numerous pitfalls to navigate: the ability to properly blind, the ethics of performing sham studies, and the practice of withholding a technology in a group of patients in whom it may be reasonably assumed could potentially benefit from its availability, to name but a few.

None of the technologies described above are perfect, with many suffering limitations unrelated to their lack of validation: namely their practicality and invasiveness in the setting of preterm neonatal care. Of those that are considered most promising within the adult population, many require insertion of arterial and venous lines to provide accurate, real-time measurements making them unsuitable as techniques in preterm infants. Among those discussed, echocardiography is likely to represent the best validated and most practical technique at present. Despite its widespread adoption, some have questioned the use of echocardiography in the NICU suggesting that it requires further validation, definition of normal values, and guidance on potential therapy before it can be optimally utilized (102). Several guidelines have been created to aid in the standardization of training and image acquisition for neonatologist-performed echocardiography (53, 103, 104). All echocardiography-based techniques have the advantage of being non-invasive and providing real-time information on blood flow. Echocardiography techniques are among the best validated in the preterm neonatal population and measurements taken can facilitate bedside decision making. Notwithstanding these advantages, echocardiography is a non-continuous measure of blood flow and although high-quality images can be obtained, potentially significant issues regarding inter-observer variability exist (65).

## Future Directions

In an editorial regarding CO monitoring in critically ill children, Chang eloquently defined three areas which warrant our focus: designing the ideal technology for CO measurement, recognition of the importance of assessing tissue perfusion, and the incorporation of non-medical expertise in the design of computer systems to analyze the increasing volumes of data which we will collect (105). The design of an ideal technology for CO monitoring will first require us to validate the existing technologies at our disposal. For some technologies such as pulmonary artery thermodilution, this is unlikely to be feasible within the preterm neonatal population. Instead we may need to validate existing technologies more rigorously around clinical outcomes and their ability to positively impact patient care; this in itself raising the question of what outcomes we should ideally be measuring. Concurrent to this, we may have to examine novel technologies more rigorously in older children and extrapolate data to the neonatal population before introducing them into practice or exploring them experimentally in premature infants. Regardless of how this is undertaken, we must appreciate that our efforts to validate existing technologies are largely to establish a relative “gold standard” within the neonatal population and that the ideal CO technology is likely to require considerable innovation and is unlikely to be closely related to any technology which is currently in use. Previous discussions on hemodynamic monitoring within the neonatal population have highlighted the importance of comprehensive hemodynamic monitoring integrating computational modeling to assist decision making (106). This idea is likely to become more important as new technologies allow us to measure cardiovascular parameters which were previously either unmeasurable or wholly unknown. Such incorporation of computer technology should be fostered in conjunction with novel CO measurements, so that as time passes and we have more information available we can make objective decisions which are most likely to benefit patient care, rather than being overwhelmed by information.

## Conclusion

There are many options available to the neonatologist to monitor CO in the preterm infants but few are well-validated within this population. There are many obstacles to creating the ideal technology to non-invasively monitor preterm CO such as the lack of a true “gold standard” within the population and the difficulty in defining true gestation-based normal ranges for a novel device. At present, echocardiography is likely to be the most robust technique available to the neonatologist though this requires experience and has a variety of limitations. Bioreactance, electrical velocimetry, and continuous Doppler measurements of CO represent the most exciting technologies under investigation due to their non-invasive nature and ability to provide continuous measurements. In the short-term, research should focus on validating existing techniques against clinical outcomes in order to best define those technologies which will impact patient care. Over the coming decades, there is need for true innovation to produce a CO technology which meets the needs of the preterm neonatal population.

## Author Contributions

MM designed the article, performed the literature review, and wrote the article. JM equally designed the article, performed the literature review, and edited article.

## Conflict of Interest Statement

The authors declare that the research was conducted in the absence of any commercial or financial relationships that could be construed as a potential conflict of interest. The handling Editor declared a past collaboration with the authors.

## Abbreviations

CO, cardiac output; ELBW, extremely low-birth weight; IVH, intraventricular hemorrhage; LVO, left ventricular output; MRI, magnetic resonance imaging; NEC, necrotizing enterocolitis; NICU, neonatal intensive care unit; PDA, patent ductus arteriosus; PICCO, pulse index continuous cardiac output; ROP, retinopathy of prematurity; RVO, right ventricular output; SVC, superior vena cava; USCOM, ultrasonic cardiac output monitor; VLBW, very low-birth weight.
